# Donor-cell leukemia with novel genetic features 2 years after sex-mismatched T cell-depleted haploidentical stem cell transplantation

**DOI:** 10.1007/s00277-020-03905-x

**Published:** 2020-02-22

**Authors:** Verena Luber, Mathias Lutz, Christian Thiede, Claudia Haferlach, Heinz Albert Dürk, Hermann Einsele, Götz Ulrich Grigoleit, Stephan Mielke

**Affiliations:** 1 Department of Internal Medicine II, University Hospital of Würzburg, Oberdürrbacher Str. 6, D-97080 Würzburg, Germany; 2 Department of Hematology and Oncology, Sana Hospital Hof, Eppenreuther Str. 9, D-95032 Hof, Germany; 3 Department of Medicine A, University Hospital of Münster, Albert-Schweitzer-Campus 1, D-48149 Münster, Germany; 4 Department of Internal Medicine I, University Hospital of Dresden Carl Gustav Carus, Fetscherstr. 74, D-01307 Dresden, Germany; 5 Munich Leukemia Laboratory, Max-Lebsche-Platz 31, D-81377 Munich, Germany; 6 Department of Hematology and Oncology, St. Josef Hospital Hamm, Albert-Struck-Str. 1, D-59075 Hamm/Bockum-Hövel, Germany; 7 Department of Laboratory Medicine and CAST, Karolinska Institutet and University Hospital, LABMED, H5, Alfred Nobels Alle 8 Plan 8, SE-141 83 Stockholm, Sweden

Dear Editor,

The mechanisms leading to the appearance of acute myeloid leukemia (AML) in the donor cells after allogeneic hematopoietic stem cell transplantation (alloHSCT) are barely understood [1, 2]. This phenomenon is associated with a poor prognosis. However, it has to be assumed that only a fraction of cases get detected and reported, making every case in its uniqueness a valuable and worthwhile to report information. While the detection of the chromosomal gender of the donor in leukemic blasts leads with certainty to the diagnosis, detection of donor cell leukemia (DCL) following sex-matched transplantation can be challenging. Here we report a well-documented case of DCL after haploidentical HSCT (haploHSCT) for secondary AML.

A 56-year-old male patient with secondary AML (karyotype 46,XY; no partial tandem duplication [PTD] of the *KMT2A [MLL]* gene) following myelodysplastic syndrome (MDS) was referred to our center for induction therapy and alloHSCT in 2010. After standard “7+3” induction chemotherapy with cytarabine and daunorubicin, the patient received conditioning with fludarabine, busulfane, and anti-thymocyte globuline (ATG) and proceeded to alloHSCT using bone marrow from a male HLA-mismatched unrelated donor. Cyclosporine and methotrexate were used as immunosuppression.

The absence of leukocyte recovery prompted us to perform bone marrow punctures at day +20 and +32 confirming primary graft-failure of unknown origin. In this life-threatening situation, it was decided to perform a rescue haploHSCT from the patient’s daughter. After reconditioning with fludarabine, cyclophosphamide, ATG, and total body irradiation (TBI), the patient received a CD34-selected peripheral blood stem cell graft. Neutrophil engraftment appeared on day +17 followed by platelet engraftment on day +50.

The post-transplant period was unspectacular showing stable hematopoiesis, donor-chimerism > 99 % in granulocytes, and between 12 and 75% in T cells and no detectable cells from the first donor.

**Fig. 1:**
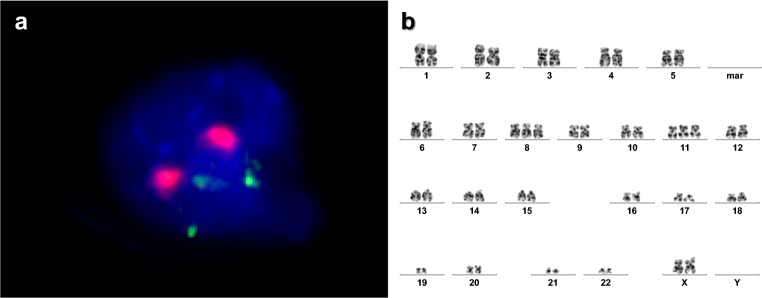
**a** Fluorescence in situ hybridization (FISH) showing one cell of the patient with two signals for centromere region of X chromosome (red color) and three signals for *KMT2A (MLL)* gene on chromosome 11 (green color). **b** Chromosome analysis of the patient demonstrating female karyotype with trisomy 8 and trisomy 11 (48,XX,+8,+11)

More than 2 years later, the patient presented with persistent fever of unknown origin. Peripheral blood count detected about 5% immature white cells so that bone marrow puncture was performed revealing 30% blasts. However, donor chimerism in peripheral blood remained almost complete with 97% and > 99% in the myeloid cells. Selected CD34^+^ cells showed also a complete donor chimerism. Moreover, FACS analysis revealed a new blastic phenotype and coinciding with this cytogenetic and molecular analysis documented novel aberrations including trisomy 8, trisomy 11, and *KMT2A*-PTD, not present at primary diagnosis of the leukemia. Chromosome banding analysis and FISH revealed the blasts to be of female origin (Fig. 1), leaving DCL as the only possible diagnosis.

The patient was reinduced with cytarabine and received conditioning therapy with cyclophosphamide, ATG, and TBI followed by a third alloHSCT from an alternative unrelated 9/10 HLA-mismatched donor. Engraftment of neutrophils was seen on day +13 and of platelets on day +12. Unfortunately, the patient developed major infective complications and died about 100 days later free of leukemia.

Development of DCL especially after haploHSCT is a poorly reported phenomenon. Just recently, a multi-center survey of the European Society for Blood and Marrow Transplantation (EBMT) estimated a frequency of 80.5 DCLs per 100,000 alloHSCTs [3]. Since proof of DCL is generally difficult to obtain, the real prevalence is probably significantly higher.

In the current case, detection of donor’s gender in leukemic blasts left no doubt as the haplo-donor was the only female donor used [4]. In other cases, leukemic rise from the first donor has indeed been described [5]. In gender-matched transplantations, it can be difficult to distinguish clonal evolution from appearance of no phenotype and/or genetic features. Indeed, about 50% of recurrent AML show different immunophenotypes and two-thirds of cases with karyotype abnormalities show changes in these abnormalities [6]. In the present case, a *KMT2A*-PTD could be detected which had not been present at primary diagnosis of AML. A previous case of DCL involving *KMT2A* gene translocation has been reported after umbilical cord blood transplantation [7].

The exact underlying mechanisms leading to DCL development remain unclear. Clonal hematopoiesis has been reported in several cases, especially from older and mostly related donors [8]. Genetic predisposition in the donor cells and effects of the recipient’s stroma and immune environment are likely to be the driving forces in DCL development. Recently, the clonal evolution in the development of DCL was described 16 months after umbilical cord blood transplantation, and similar to the present case, no genetic alterations were found in the donor cells before transplantation [9]. The application of G-CSF to the donor cannot be linked to this development but maintains a hypothetical possibility in a pre-existing condition. Although there is an association between donor and host features in 80% of DCL cases [10], it has to be assumed that only a minority of donors will develop leukemia or MDS as anecdotal case reports are rare [8]. In the present case, we performed a bone marrow puncture on the daughter after obtaining informed consent. Morphological, cytogenetical, and mutational diagnostics revealed normal hematopoiesis with normal karyotype (46,XX) and no *KMT2A*-PTD, no *FLT3*-ITD, no *FLT3*-TKD, and no mutations of *ASXL1*, *CBL*, *CEBPA*, *IDH1*R132, *IDH2*, *KIT*, *KRAS*, *NPM1*, *NRAS*, *RUNX1*, *TET2*, *TP53*, and *WT1*. Next-generation sequencing was not performed at that time. However, the absence of *KMT2A*-PTD made pre-existing occult leukemia in the donor cells unlikely. In the current case, the lack of lymphocytes in the CD34-selected haploidentical stem cell allograft may have indeed hampered the immune surveillance of rising genetic instability. Nowadays, CD34-selected haploidentical stem cell sources which are more likely to be followed by adoptive transfer of selected or manipulated lymphocytes or T-replete procedures such us post-transplant cyclophosphamide are applied [11].

Taken together, it appears to remain important to inform the readership continuously about the barely understood phenomenon of DCL development alerting everybody to consider the rare in situations when relapse is present in context of full donor chimerism.
